# Enchentes no Rio Grande do Sul, Brasil, em 2024, e seus efeitos na epidemiologia da leptospirose

**DOI:** 10.1590/0102-311XPT111125

**Published:** 2026-07-06

**Authors:** Bernardo Madeira Diefenthaeler, Alexia Antunes Deluca, Jéssica Manami Seki, Marco Antônio Tavares Duarte, Vinícius da Silva Gregory, Sofia Giacomet Borges, Roger Ferreira Gomes, Maria Helena Rigatto

**Affiliations:** 1 Faculdade de Medicina, Universidade Federal do Rio Grande do Sul, Porto Alegre, Brasil.; 2 Faculdade de Odontologia, Universidade Federal do Rio Grande do Sul, Porto Alegre, Brasil.; 3 Universidade Federal de Ciências da Saúde de Porto Alegre, Porto Alegre, Brasil.; 4 Programa de Pós-graduação em Ciências Médicas, Universidade Federal do Rio Grande do Sul, Porto Alegre, Brasil.

**Keywords:** Leptospirose, Inundações, Doenças de Veiculação Hídrica, Leptospirosis, Floods, Waterborne Diseases, Leptospirosis, Inundaciones, Enfermedades de Vehiculación Hídrica

## Abstract

Eventos climáticos extremos favorecem a disseminação de doenças de veiculação hídrica. Em 2024, o Rio Grande do Sul, Brasil, enfrentou enchentes históricas, cujo efeito sobre a ocorrência de leptospirose foi analisado neste estudo. Trata-se de um estudo transversal descritivo, realizado com dados secundários de sistemas oficiais de saúde, comparando casos confirmados, internações e óbitos entre abril e julho de 2023 e de 2024. Foram examinadas variações mensais, características sociodemográficas e distribuição espacial dos casos. Considerando o período de abril a julho, o número de casos aumentou de 117 em 2023 para 1.007 em 2024, um crescimento de 7,6 vezes, com pico sendo registrado em maio de 2024 (516 registros). As internações passaram de 89 para 228, destacando-se junho (76 em 2024 contra 15 em 2023), e os óbitos subiram de 7 para 35, com maior aumento correspondendo ao mês de maio (1 em 2023 e 20 em 2024). Apesar do aumento absoluto, a letalidade caiu de 6% em 2023 para 3,5% em 2024. Os grupos mais afetados em 2024 permaneceram homens jovens e brancos na relação com 2023. Mais de 200 municípios notificaram casos em 2024, com destaque para Porto Alegre, Alvorada, Canoas e São Leopoldo (todos integrantes da Região Metropolitana de Porto Alegre), que concentraram quase metade das ocorrências. Os resultados sugerem que as enchentes contribuem para a intensificação da transmissão, a sobrecarga hospitalar e o aumento dos óbitos por leptospirose, reforçando a importância da integração entre a vigilância epidemiológica, a infraestrutura urbana e as estratégias preventivas, a fim de reduzir a vulnerabilidade populacional diante de futuros desastres climáticos.

## Abstract (en)

Extreme climate events favor the spread of waterborne diseases. This study analyzes the impact of the historic floods occurred in Rio Grande do Sul State, Brazil, in 2024 on leptospirosis incidence. This is a descriptive cross-sectional study based on secondary data from official health information systems, comparing confirmed cases, hospitalizations, and deaths between April and July 2023 and the same period in 2024. Monthly trends, sociodemographic characteristics, and spatial distribution of cases were examined. From April to July, the number of cases increased from 117 in 2023 to 1,007 in 2024, a 7.6-fold rise, with a peak in May 2024 (516 cases). Hospitalizations increased from 89 to 228, with June showing a marked rise (76 in 2024 and 15 in 2023), and deaths rose from seven to 35, with the sharpest increase in May (one in 2023 and 20 in 2024). Despite the absolute increase, mortality rates declined from 6% in 2023 to 3.5% in 2024. In 2024, the most affected groups remained young white men, consistent with the 2023 profile. Over 200 municipalities reported cases in 2024, particularly Porto Alegre, Alvorada, Canoas, and São Leopoldo (all located in the Porto Alegre Metropolitan Area), which together accounted for nearly half of cases. The findings suggest that floods contributed to intensified transmission, increased hospital burden, and higher leptospirosis-related mortality. These results underscore the need for integrated epidemiological surveillance, resilient urban infrastructure, and preventive strategies to reduce population vulnerability to future climate-related disasters.

## Abstract (es)

Los fenómenos climáticos extremos favorecen la propagación de enfermedades transmitidas por el agua. En 2024, el estado de Rio Grande do Sul, Brasil, sufrió inundaciones históricas, y en este estudio se analizó su efecto sobre la incidencia de la leptospirosis. Se trata de un estudio transversal descriptivo, realizado con datos secundarios de sistemas oficiales de salud, en el que se comparan los casos confirmados, las hospitalizaciones y las muertes entre abril y julio de 2023 y de 2024. Se examinaron las variaciones mensuales, las características sociodemográficas y la distribución espacial de los casos. Teniendo en cuenta el periodo comprendido entre abril y julio, el número de casos aumentó de 117 en 2023 a 1.007 en 2024, lo que supone un incremento de 7,6 veces, con un pico registrado en mayo de 2024 (516 registros). Las hospitalizaciones pasaron de 89 a 228, destacando el mes de junio (76 en 2024 frente a 15 en 2023), y las muertes aumentaron de 7 a 35, con el mayor incremento correspondiente al mes de mayo (1 en 2023 y 20 en 2024). A pesar del aumento absoluto, la letalidad bajó del 6% en 2023 al 3,5% en 2024. Los grupos más afectados en 2024 siguieron siendo los hombres jóvenes y blancos, en relación con 2023. Más de 200 municipios notificaron casos en 2024, destacando Porto Alegre, Alvorada, Canoas y São Leopoldo (todos ellos pertenecientes a la Región Metropolitana de Porto Alegre), que concentraron casi la mitad de los casos. Los resultados sugieren que las inundaciones contribuyen a la intensificación de la transmisión, la sobrecarga hospitalaria y el aumento de las muertes por leptospirosis, lo que refuerza la importancia de la integración entre la vigilancia epidemiológica, la infraestructura urbana y las estrategias preventivas, con el fin de reducir la vulnerabilidad de la población ante futuros desastres climáticos.

## Introdução

Eventos climáticos extremos, como enchentes, ondas de calor e furacões, representam riscos crescentes à saúde global ^1^. Embora possam ocorrer naturalmente, a mudança climática ‒ marcada pelo aumento da temperatura média e por padrões de precipitação alterados ‒ vem ampliando a frequência e a severidade destes ^1^. Entre seus efeitos adversos, destaca-se o incremento do risco de zoonoses ^2^, sobretudo a leptospirose, cuja transmissão é favorecida por enchentes que ampliam a dispersão ambiental da bactéria e a exposição humana ^3^.

A leptospirose é uma zoonose de relevância epidemiológica, causada por espiroquetas do gênero *Leptospira*, sendo a *Leptospira interrogans* a mais frequentemente implicada em casos humanos ^4^. O quadro clínico varia de formas febris autolimitadas a manifestações graves, como a síndrome de Weil e a hemorragia pulmonar, associadas a elevada letalidade ^4^.

A transmissão ocorre pelo contato da pele ou por mucosas através da água ou do solo contaminados pela urina de animais infectados, principalmente roedores ^4^ ^,^ ^5^. Em contextos de enchentes, a disseminação da bactéria em ambientes urbanos se intensifica, ampliando os pontos de exposição e dificultando medidas preventivas. A circulação inevitável em áreas alagadas aumenta rapidamente a exposição populacional e, consequentemente, o risco de infecção ^3^.

A ocorrência da doença, entretanto, não pode ser explicada apenas pela exposição ambiental. Determinantes sociais e urbanos, como saneamento precário, habitação em áreas de risco, manejo inadequado de resíduos e vulnerabilidade socioeconômica, exercem papel central na dinâmica de transmissão, evidenciando a influência dos fatores sociais na distribuição da doença ^5^ ^,^ ^6^. Estudos apontam maior acometimento entre homens jovens e populações urbanas socialmente marginalizadas ^5^ ^,^ ^7^.

Territórios urbanos vulneráveis, caracterizados por saneamento inadequado, esgoto a céu aberto e acúmulo de resíduos, apresentam maior risco de transmissão em razão da elevada densidade de roedores e do maior contato humano com fontes de contaminação ^6^ ^,^ ^8^. Essas vulnerabilidades tendem a ser intensificadas durante eventos hidrometeorológicos extremos, afetando de forma desproporcional populações residentes em áreas sujeitas a alagamentos e com menor capacidade de resposta. Nesses contextos, a sobreposição de perdas materiais, desabrigo e adoecimento ‒ incluindo a leptospirose ‒ evidencia o peso das desigualdades sociais e territoriais na ocorrência dessa zoonose ^9^ ^,^ ^10^.

Epidemiologicamente, a leptospirose é reconhecida como uma das zoonoses mais disseminadas no mundo, com estimativas de 1,03 milhão de casos e 58.900 óbitos anuais, sobretudo em países tropicais de baixa e média renda ^4^ ^,^ ^11^. No Brasil, trata-se de um agravo de notificação compulsória, com média anual de 3.400 casos e letalidade de 9% ^12^ ‒ valor superior à média global de 5,72% ^11^ e à média de 5% observada em surtos internacionais ^13^.

No Brasil, as regiões Sul e Sudeste concentram a maior parte dos registros de leptospirose ^14^. No Rio Grande do Sul, a média é de 477,4 casos anuais, com incidência quatro vezes superior à média nacional ^15^. A Região Metropolitana de Porto Alegre, principal aglomeração urbana do estado, responde por 46,3% dos casos confirmados ^14^. Além disso, indivíduos entre 20 e 59 anos correspondem a 56% dos registros ^14^.

O Rio Grande do Sul apresenta histórico recorrente de enchentes associadas a chuvas intensas e à elevação súbita dos níveis dos rios ^16^, com intensificação e aumento da frequência desses eventos nas últimas décadas, em consonância com as tendências globais das mudanças climáticas ^1^ ^,^ ^17^. Em setembro de 2023, inundações atingiram mais de 100 municípios, causando óbitos e deslocamentos populacionais ^17^. Em abril e maio de 2024, chuvas persistentes provocaram a maior enchente da história estadual, afetando cerca de 95% dos municípios, especialmente a Região Metropolitana de Porto Alegre e os vales ribeirinhos, com decretação de calamidade pública e ampla exposição ambiental ^17^ ^,^ ^18^. Nesse contexto, era esperado o aumento de doenças de veiculação hídrica, como a leptospirose, conforme antecipado em boletins oficiais e estudos recentes ^19^ ^,^ ^20^ ^,^ ^21^.

Apesar de sua ampla distribuição e elevada letalidade, a leptospirose ainda é uma doença negligenciada, com investimentos insuficientes em prevenção, vigilância e pesquisa, especialmente em países de baixa e média renda. Essa negligência estrutural sustenta lacunas na capacidade de resposta, resultando em subdiagnóstico, diagnóstico tardio e limitações no controle da doença ^10^, particularmente evidentes no Brasil durante desastres climáticos, quando o aumento da demanda assistencial expõe fragilidades persistentes em áreas vulneráveis ^9^.

Apesar de sua relevância epidemiológica, são escassos os estudos que integram análises temporais e espaciais da leptospirose em contextos de eventos hidrometeorológicos extremos, limitando a compreensão da magnitude e da dinâmica do problema, especialmente em regiões vulneráveis como o Sul do Brasil ^5^. Até o momento, não foram identificadas investigações sobre as enchentes de 2024 que descrevam, de forma integrada, casos confirmados, hospitalizações e óbitos ^19^ ^,^ ^20^ ^,^ ^21^. Ademais, grande parte dos estudos anteriores não contemplou integralmente o mês de abril ‒ caracterizado por intensas precipitações ‒, o que pode ter resultado na subestimação dos efeitos iniciais ^19^ ^,^ ^20^.

Diante desse cenário, este estudo objetiva descrever os casos confirmados, as internações e os óbitos por leptospirose humana de abril a julho de 2024 no Rio Grande do Sul, comparando-os ao mesmo período de 2023. Busca-se caracterizar a variação temporal, o perfil sociodemográfico e a distribuição espacial dos casos, de modo a contribuir para a compreensão da relação entre desastres climáticos extremos e a ocorrência da doença.

## Métodos

Trata-se de um estudo transversal descritivo, baseado em dados secundários de domínio público. As informações sobre casos confirmados e óbitos por leptospirose foram obtidas no Sistema de Informação de Agravos de Notificação (SINAN) ^22^, e os dados de internações no Sistema de Informações Hospitalares do Sistema Único de Saúde (SIH/SUS) ^23^, ambos acessados pela plataforma TabNet do Departamento de Informação e Informática do SUS (DATASUS) em 22 de setembro de 2025.

O estudo foi conduzido no Estado do Rio Grande do Sul, composto por 497 municípios, com área territorial de 281,7 mil km^2^. O estado apresenta população de 10,9 milhões de habitantes ‒ predominantemente urbana (87,5%) ‒ e Índice de Desenvolvimento Humano (IDH) de 0,771 ^24^ ^,^ ^25^.

O território do estado apresenta clima subtropical, com temperaturas médias anuais entre 15ºC e 18ºC, precipitação anual de 1.200 a 1.800mm e extensa rede hidrográfica composta de 25 bacias, organizadas em três Regiões Hidrográficas: Rio Uruguai, Guaíba e Bacias Litorâneas. A Região Hidrográfica do Guaíba concentra as principais áreas urbanas e industriais do estado, incluindo os vales dos rios Taquari, Caí e Baixo Jacuí ^25^.

Durante os eventos climáticos extremos de abril e maio de 2024, mais de 90% do território estadual foi afetado, com decretação de calamidade pública em 78 municípios e situação de emergência em outros 340 até 31 de maio. Entre as áreas mais impactadas, destacaram-se a Região Metropolitana de Porto Alegre e os vales dos rios Taquari, Caí e Pardo, integrantes da Região Hidrográfica do Guaíba ^18^ ^,^ ^26^. Para este estudo, foram considerados todos os 497 municípios do Rio Grande do Sul.

O recorte temporal considerou: (1) o período de inundação (24 de abril a 31 de maio de 2024) ^27^; e (2) o período de incubação da leptospirose (1 a 30 dias, com média de 5 a 14 dias) ^28^. Dessa forma, foram analisados os meses de abril a julho de 2024, comparados ao mesmo período de 2023. Abril foi incluído por marcar o início das enchentes, quando casos com incubação curta já poderiam ser notificados, enquanto julho foi considerado por possíveis períodos de incubação prolongada e notificações tardias. Embora a calamidade pública tenha sido decretada em 1º de maio de 2024 (*Decreto Estadual nº 57.596*) e prorrogada posteriormente ^27^, adotou-se como referência o período da inundação e o intervalo esperado para manifestação da leptospirose.

Os casos confirmados foram agregados por mês e analisados quanto à taxa de incidência ‒ expressa em casos por 100 mil habitantes ‒, calculada com base na população de 10.882.665 habitantes estimada pelo *Censo Demográfico* de 2022 do Instituto Brasileiro de Geografia e Estatística (IBGE) ^24^. As internações foram extraídas do TabNet/DATASUS, considerando internações ocorridas no Rio Grande do Sul, nos períodos de abril a julho de 2023 e de 2024, segundo os códigos da 10ª revisão da Classificação Internacional de Doenças (CID-10): A27.0 (Infecção por *Leptospira icterohaemorrhagiae*), A27.8 (Outras formas de leptospirose) e A27.9 (Leptospirose não especificada), conforme a Tabela de Morbidade Hospitalar do SIH/SUS. Os casos confirmados e os óbitos foram obtidos da plataforma TabNet/DATASUS, por meio do banco “Doenças e Agravos de Notificação ‒ 2007 em diante (SINAN)”, selecionando-se leptospirose, o Estado do Rio Grande do Sul e os meses de abril a julho de 2023 e de 2024. Para os óbitos, foram incluídos apenas os registros com evolução classificada como “óbito pelo agravo notificado”.

Adicionalmente, foram estimados indicadores derivados, incluindo taxa de letalidade (óbitos/casos confirmados × 100), variação absoluta entre 2024 e 2023 e a razão 2024/2023 para cada desfecho. Também foi calculada a proporção dos registros do quadrimestre em relação ao total anual de casos.

A caracterização sociodemográfica considerou idade (0-19, 20-39, 40-59, 60-79 e ≥ 80 anos), sexo, raça/cor e escolaridade. Os dados foram agrupados por ano (2023 e 2024), estratificados por desfecho (casos, internações e óbitos) e apresentados em valores absolutos e relativos. As internações não foram estratificadas por escolaridade, por indisponibilidade dessa variável no SIH/SUS. Para padronização etária, as categorias originalmente detalhadas foram agrupadas nas faixas adotadas.

Os casos de leptospirose incluídos corresponderam à classificação “confirmado” no SINAN. Segundo o Ministério da Saúde, um caso é confirmado quando apresenta quadro clínico compatível associado à confirmação laboratorial, como soroconversão, aumento de quatro vezes no título da prova de microaglutinação (MAT), isolamento em cultura, detecção de DNA por PCR ou título único elevado em MAT. Na ausência de confirmação laboratorial, admite-se a confirmação por critério clínico-epidemiológico, considerando sinais e sintomas típicos associados à exposição a ambientes de risco, como enchentes, água ou lama contaminada, atividades ocupacionais de risco ou vínculo epidemiológico, desde que não haja diagnóstico alternativo. A classificação final é atribuída pela vigilância municipal ou estadual ^28^. Este estudo também comparou a distribuição dos critérios de confirmação entre 2023 e 2024, considerando o período de abril a julho.

**Figura 1:**
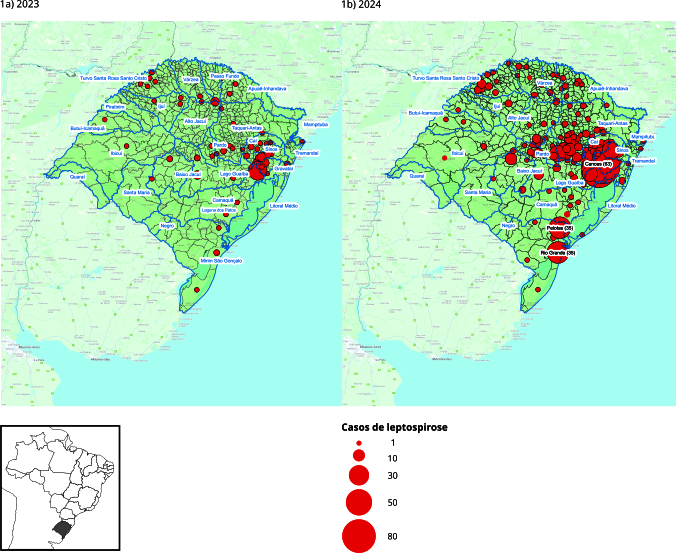
Distribuição espacial dos casos confirmados de leptospirose no Rio Grande do Sul, Brasil, por município, entre abril e julho de 2023 e de 2024.

Adicionalmente, foi realizada uma análise espacial descritiva dos casos confirmados de leptospirose por município de notificação, referentes aos períodos de abril a julho de 2023 e de 2024 no Rio Grande do Sul. Os mapas (Figura 1) foram elaborados no QGIS 3.34 (https://qgis.org/), com base nos limites municipais do IBGE. As bacias hidrográficas da Agência Nacional de Águas e Saneamento Básico (ANA) foram incluídas para relacionar a distribuição dos casos às áreas afetadas pelas cheias. Os casos foram representados por círculos proporcionais ao número de registros, com identificação nominal dos municípios com mais de 30 casos. Não foram aplicados métodos estatísticos adicionais, tratando-se de análise descritiva da distribuição geográfica.

Fonte: elaborada utilizando o software QGIS, versão 3.34. Os dados de casos confirmados de leptospirose (abril a julho de 2023 e de 2024) foram obtidos no Sistema de Informações de Agravos de Notificação (SINAN), acessados via Departamento de Informação e Informática do SUS (DATASUS) em 22 de setembro de 2025 ^22^. A base cartográfica municipal foi disponibilizada pelo Instituto Brasileiro de Geografia e Estatística (IBGE), e a delimitação das bacias hidrográficas foi obtida junto à Agência Nacional de Águas e Saneamento Básico (ANA).

Os dados foram processados no Google Sheets (https://docs.google.com/spreadsheets/create), utilizando funções básicas para o cálculo de incidência, indicadores derivados e proporções. Todas as informações utilizadas são de acesso público, obtidas em plataformas oficiais do DATASUS ^22^ ^,^ ^23^, assegurando rastreabilidade e reprodutibilidade. Por se tratar de dados secundários e públicos, o estudo está dispensado de apreciação por comitê de ética em pesquisa, conforme as *Resoluções nº 466/2012* e *nº 510/2016* do Conselho Nacional de Saúde.

## Resultados

### Caracterização sociodemográfica dos desfechos

A Tabela 1 apresenta a distribuição dos casos confirmados, internações e óbitos por leptospirose segundo idade, sexo, raça/cor e escolaridade. Não foram observadas variações relevantes nas categorias escolaridade e raça/cor entre 2023 e 2024, mantendo-se o predomínio de pessoas autodeclaradas brancas. Quanto ao sexo, verificou-se leve aumento proporcional do acometimento feminino em 2024, embora o predomínio masculino permaneça em todos os desfechos. Em relação à idade, observou-se maior concentração entre adultos jovens (20-39 anos) e de meia-idade (40-59 anos), com aumento proporcional nesta última faixa etária.

**Tabela 1:** Distribuição dos casos confirmados, internações e óbitos por leptospirose segundo faixa etária, sexo, raça/cor e escolaridade no Rio Grande do Sul, Brasil, nos períodos entre abril e julho de 2023 e de 2024.

Variáveis	2023			2024		
	Casos confirmados	Internações	Óbitos	Casos confirmados	Internações	Óbitos
	n (%)	n (%)	n (%)	n (%)	n (%)	n (%)
Idade (anos)						
0-19	16 (13,7)	9 (10,1)	0 (0,0)	78 (7,7)	21 (9,2)	0 (0,0)
20-39	48 (41,0)	36 (40,4)	3 (42,9)	420 (41,7)	73 (32,0)	7 (20,0)
40-59	34 (29,1)	25 (28,1)	0 (0,0)	388 (38,5)	90 (39,5)	18 (51,4)
60-79	17 (14,5)	17 (19,1)	3 (42,9)	116 (11,5)	42 (18,4)	9 (25,7)
≥ 80	2 (1,7)	2 (2,2)	1 (14,3)	5 (0,5)	2 (0,9)	1 (2,9)
Sexo						
Masculino	84 (71,8)	76 (85,4)	6 (85,7)	707 (70,2)	180 (78,9)	30 (85,7)
Feminino	33 (28,2)	13 (14,6)	1 (14,3)	300 (29,8)	48 (21,1)	5 (14,3)
Raça/Cor						
Amarela	1 (0,9)	1 (1,1)	0 (0,0)	5 (0,5)	3 (1,3)	0 (0,0)
Branca	87 (74,4)	79 (88,8)	7 (100,0)	765 (76,0)	192 (84,2)	26 (74,3)
Parda	15 (12,8)	2 (2,2)	0 (0,0)	65 (6,5)	17 (7,5)	4 (11,4)
Preta	9 (7,7)	6 (6,7)	0 (0,0)	64 (6,4)	16 (7,0)	2 (5,7)
Ignorado/Sem informação	5 (4,3)	1 (1,1)	0 (0,0)	108 (10,7)	0 (0,0)	3 (8,6)
Escolaridade						
Analfabeto	1 (0,9)	-	0 (0,0)	1 (0,1)	-	0 (0,0)
1ª a 4ª série incompleta do Ensino Fundamental	6 (5,1)	-	0 (0,0)	26 (2,6)	-	3 (8,6)
4ª série completa do Ensino Fundamental	5 (4,3)	-	1 (14,3)	28 (2,8)	-	2 (5,7)
5ª a 8ª série incompleta do Ensino Fundamental	15 (12,8)	-	1 (14,3)	65 (6,5)	-	2 (5,7)
Ensino Fundamental completo	9 (7,7)	-	0 (0,0)	67 (6,7)	-	3 (8,6)
Ensino Médio incompleto	13 (11,1)	-	1 (14,3)	40 (4,0)	-	2 (5,7)
Ensino Médio completo	15 (12,8)	-	1 (14,3)	134 (13,3)	-	4 (11,4)
Educação Superior incompleta	4 (3,4)	-	0 (0,0)	17 (1,7)	-	0 (0,0)
Educação Superior completa	5 (4,3)	-	0 (0,0)	29 (2,9)	-	0 (0,0)
Não se aplica	1 (0,9)	-	0 (0,0)	12 (1,2)	-	0 (0,0)
Ignorado/sem informação	43 (36,8)	-	3 (42,9)	588 (58,4)	-	19 (54,3)
Total	117 (100,0)	89 (100,0)	7 (100,0)	1.007 (100,0)	228 (100,0)	35 (100,0)

Fonte: os dados foram obtidos no Sistema de Informações de Agravos de Notificação (SINAN) e no Sistema de Informações Hospitalares do Sistema Único de Saúde (SIH/SUS), acessados via Departamento de Informação e Informática do SUS (DATASUS) em 22 de setembro de 2025 ^22^ ^,^ ^23^.

### Incidência de leptospirose

Entre abril e julho, foram registrados 117 casos confirmados de leptospirose em 2023 e 1.007 em 2024 (Tabela 2). O aumento absoluto foi de 890 casos, sendo o número de registros em 2024 aproximadamente 7,6 vezes maior que em 2023. O pico ocorreu em maio de 2024, com 516 casos e incidência de 4,7 por 100 mil habitantes. O maior incremento relativo também foi observado em maio (15,6 vezes superior a 2023), seguido por junho (9,3 vezes) e julho (3,1 vezes). Em 2023, os 117 casos corresponderam a 25,3% do total anual (462), enquanto em 2024, os 1.007 casos representaram 75,5% do total (1.333).

**Tabela 2:** Comparação de casos confirmados e incidência relacionada à leptospirose no Rio Grande do Sul, Brasil, nos períodos entre abril e julho de 2023 e de 2024.

Mês	2023		2024		Aumento absoluto de casos	Razão 2024/2023
	Casos confirmados	Incidência (por 100.000 habitantes)	Casos confirmados	Incidência (por 100.000 habitantes)		
Abril	25	0,23	55	0,51	30	2,20
Maio	31	0,28	516	4,74	485	16,65
Junho	30	0,28	310	2,85	280	10,33
Julho	31	0,28	126	1,16	95	4,06
Total	117	1,08	1.007	9,25	890	8,61

Fonte: os dados foram obtidos no Sistema de Informações de Agravos de Notificação (SINAN), acessados via Departamento de Informação e Informática do SUS (DATASUS) em 22 de setembro de 2025 ^22^. A população do Rio Grande do Sul utilizada para o cálculo de incidência foi de 10.882.665 habitantes - seguindo a estimativa do *Censo Demográfico* de 2022 do Instituto Brasileiro de Geografia e Estatística (IBGE) ^24^.

### Internações por leptospirose

As internações entre abril e julho aumentaram de 89, em 2023, para 228, em 2024, acréscimo absoluto de 139 registros, correspondendo a aproximadamente 1,6 vez mais hospitalizações. O maior incremento mensal ocorreu em junho, com 76 internações em 2024 frente a 15 em 2023, cerca de quatro vezes mais (Tabela 3).

**Tabela 3:** Comparação das internações relacionadas à leptospirose no Rio Grande do Sul, Brasil, nos períodos entre abril e julho de 2023 e de 2024.

Mês	Internações		Variação absoluta de internações	Razão 2024/2023
	2023	2024		
Abril	24	23	-1	0,96
Maio	22	63	41	2,86
Junho	15	76	61	5,07
Julho	28	66	38	2,36
Total	89	228	139	2,56

Fonte: os dados foram obtidos no Sistema de Informações Hospitalares do Sistema Único de Saúde (SIH/SUS), acessados via Departamento de Informação e Informática do SUS (DATASUS) em 22 de setembro de 2025 ^23^.

### Óbitos por leptospirose

Os óbitos por leptospirose no quadrimestre aumentaram de 7, em 2023, para 35, em 2024, variação absoluta de 28, correspondendo a um acréscimo de quatro vezes. O maior número de mortes concentrou-se em maio, com 20 óbitos em 2024 frente a apenas um em 2023, aumento de 19 vezes. A Tabela 4 apresenta as taxas de letalidade por mês e ano. Apesar do aumento absoluto de mortes em 2024, a letalidade global do quadrimestre foi menor (3,5%) comparada a 2023 (6%), indicando que o crescimento dos casos confirmados foi proporcionalmente maior que o de óbitos.

**Tabela 4:** Comparação dos óbitos relacionados à leptospirose no Rio Grande do Sul, Brasil, nos períodos entre abril e julho de 2023 e de 2024.

Mês	2023		2024		Variação absoluta de óbitos	Razão 2024/2023
	Óbitos	Letalidade (%)	Óbitos	Letalidade (%)		
Abril	2	8	6	10,91	4	3
Maio	1	3,23	20	3,88	19	20
Junho	0	0	7	2,26	7	*
Julho	4	12,90	2	1,59	-2	0,5
Total	7	5,98	35	3,48	28	5

Fonte: os dados foram obtidos no Sistema de Informações de Agravos de Notificação (SINAN), acessados via Departamento de Informação e Informática do SUS (DATASUS) em 22 de setembro de 2025 ^22^.

* Razão não calculada porque não houve óbitos em junho de 2023 (denominador zero).

### Critério confirmatório dos casos

Ao comparar os critérios de confirmação registrados no SINAN entre abril e julho, observou-se que, em 2023, dos 117 casos confirmados, a maioria foi classificada como clínico-laboratorial (100; 85,5%), enquanto 15 (12,8%) foram clínico-epidemiológicos e 2 (1,7%) constavam como ignorado/sem informação. Em 2024, contudo, verificou-se uma mudança expressiva nesse perfil: entre os 1.007 casos confirmados, 499 (49,6%) foram classificados como clínico-laboratoriais, 498 (49,5%) como clínico-epidemiológicos e 10 (1%) como ignorado/sem informação.

### Distribuição espacial dos casos

A análise espacial evidenciou um forte contraste entre 2023 e 2024 (Figura 1). Em 2023, os casos concentraram-se em poucos municípios, com destaque para Porto Alegre, que registrou 26 ocorrências. Em 2024, observou-se uma expansão expressiva, com mais de 200 municípios apresentando casos confirmados, concentrados nas áreas metropolitanas e ribeirinhas. Porto Alegre contabilizou 246 casos (24,4% do total estadual), um aumento absoluto de 220 em relação a 2023. Outros municípios da Região Metropolitana de Porto Alegre também tiveram crescimento, como Alvorada (83 casos; 8,2%), Canoas (63; 6,3%) e São Leopoldo (48; 4,8%). Em conjunto, esses quatro municípios responderam por 43,7% dos registros de 2024.

Municípios que registraram poucos ou nenhum caso em 2023 apresentaram aumento em 2024, como Três Coroas (42; 4,2%), Igrejinha (22; 2,2%), Nova Santa Rita (18; 1,8%) e Guaíba (17; 1,7%). No interior do estado, destacaram-se Pelotas e Rio Grande, ambos com 35 casos (3,5% cada), além de Santa Cruz do Sul (20) e Santa Maria (10). Esse padrão evidenciou maior impacto na Região Metropolitana de Porto Alegre, nos vales dos rios Taquari e Caí e no litoral sul, áreas coincidentes com aquelas afetadas pelas enchentes.

## Discussão

O aumento dos casos de leptospirose no Rio Grande do Sul em 2024 configura relevante problema de saúde pública, associado às condições ambientais decorrentes das enchentes, que ampliaram a exposição da população a águas contaminadas. O crescimento de casos, internações e óbitos sugere o impacto direto do desastre climático na transmissão da doença, em consonância com evidências de outras regiões, onde eventos hidrometeorológicos extremos foram seguidos por elevação significativa da incidência da doença ^4^ ^,^ ^29^ ^,^ ^30^. Durante o período das cheias no Rio Grande do Sul, foram registrados 1.007 casos (7,6 vezes mais que em 2023) e 35 óbitos (quatro vezes superior a 2023).

A associação entre precipitação elevada e aumento da leptospirose é amplamente documentada ^4^ ^,^ ^31^. Em Manila (Filipinas), enchentes decorrentes de um tufão em 2009 resultaram em 2.299 casos suspeitos e 178 óbitos ^32^. No Brasil, estudos no Paraná e em Santa Catarina também demonstraram correlação entre picos de precipitação e notificações ^31^ ^,^ ^33^. No Rio Grande do Sul, Ranieri et al. ^19^ relataram aumento da incidência e da mortalidade após as enchentes de 2024, reforçando a relevância epidemiológica do evento. Embora ambos os estudos utilizem dados abertos do Ministério da Saúde, diferenças metodológicas explicam as variações observadas. Ranieri et al. ^19^ analisaram bases consolidadas do SINAN estadual no período de maio a julho, enquanto este estudo utilizou dados do SINAN nacional no período de abril a julho ‒ abrangendo o início das enchentes em abril, mês que apresentou um aumento de 30 casos em relação a 2023 (incremento de 120%). Ademais, o estudo incorporou variáveis adicionais, como hospitalizações, escolaridade, raça/cor e a distribuição dos casos por bacias hidrográficas, ampliando a análise da carga da doença.

O aumento das hospitalizações por leptospirose em 2024, cerca de 1,6 vez em relação a 2023, reforça a gravidade do cenário, uma vez que a necessidade de internação indica maior ocorrência de formas clínicas graves. Evidências prévias mostram que as enchentes ampliam não apenas a transmissão, mas também a pressão sobre os serviços hospitalares ^34^.

De modo semelhante, a mortalidade apresentou crescimento expressivo, com 35 óbitos ‒ quatro vezes mais que em 2023 ‒ valor próximo ao observado por Ranieri et al. ^19^, que relataram aumento de cinco vezes. Essa convergência reforça a associação entre desastres climáticos e desfechos fatais.

Os aumentos nas hospitalizações e nos óbitos por leptospirose indicam que as enchentes, além de intensificarem a transmissão, podem agravar a evolução clínica dos casos. Esse cenário representa desafio crítico para o Sistema Único de Saúde (SUS), especialmente porque eventos climáticos extremos também elevam hospitalizações e mortalidade por outros agravos ‒ doenças cardiovasculares, respiratórias, infecciosas, digestivas e por transtornos mentais ‒ ampliando o risco de sobrecarga e potencial colapso dos serviços de saúde ^35^.

Os achados contribuem para subsidiar estratégias de organização da rede assistencial em futuros desastres climáticos, destacando a importância do diagnóstico precoce, do acesso oportuno à antibioticoterapia e do fortalecimento da capacidade hospitalar durante emergências ambientais ^36^.

Durante as enchentes, as autoridades estaduais adotaram recomendações técnicas de manejo clínico, adaptações nos protocolos de vigilância e ações preventivas ^37^ ^,^ ^38^ ^,^ ^39^. No âmbito assistencial, orientou-se o início imediato da antibioticoterapia em casos suspeitos, respaldado pela elevada letalidade das formas graves e pela maior eficácia do tratamento precoce ^39^. Na vigilância, os protocolos foram flexibilizados, permitindo o encerramento de casos por critério clínico-epidemiológico ou por resultados de laboratórios privados, diante da inviabilidade temporária de envio de amostras ao Laboratório Central do Rio Grande do Sul (LACEN) ^37^ ^,^ ^38^. Essa mudança reflete-se nos seguintes resultados: entre abril e julho de 2023, 12,8% dos casos foram confirmados por critério clínico-epidemiológico, proporção que aumentou para 49,5% em 2024. No campo da prevenção, destacaram-se orientações sobre uso de equipamentos de proteção individual, cobertura de ferimentos, higiene rigorosa, desinfecção ambiental com hipoclorito e atualização da vacinação antitetânica ^37^ ^,^ ^39^.

As medidas adotadas estão alinhadas às recomendações do Ministério da Saúde para contextos de inundações, que enfatizam estratégias de detecção, monitoramento e resposta ao risco de leptospirose ^40^. As diretrizes reforçam a divulgação de informações à população exposta, a notificação de todos os casos suspeitos no SINAN e a comunicação integrada entre vigilância e serviços de atenção primária e especializada ^40^.

Contudo, a experiência durante as enchentes de 2024 evidenciou fragilidades estruturais que limitaram a resposta institucional. A sobrecarga abrupta de casos, associada à indisponibilidade de testes rápidos e à necessidade de envio de amostras a laboratórios de referência, gerou atrasos diagnósticos. Além disso, a interrupção de serviços essenciais, como energia, comunicação e internet, comprometeu temporariamente o sistema oficial de notificação, exigindo mecanismos provisórios paralelos de vigilância e possivelmente afetando a completude dos dados epidemiológicos ^19^.

Esses desafios indicam que, embora existam normas e protocolos, a capacidade de resposta do SUS a crises de grande escala depende também de infraestrutura adequada, logística laboratorial eficiente, mobilização rápida e sistemas de vigilância resilientes, frequentemente fragilizados em desastres socioambientais, sobretudo em territórios vulneráveis.

O estudo identificou predominância de casos em homens (70,2%) e na faixa etária de 20 a 39 anos (41,7%), perfil consistente com a literatura, que aponta maior risco entre homens jovens expostos a atividades ocupacionais ou de resgate durante enchentes ^3^ ^,^ ^4^ ^,^ ^5^ ^,^ ^11^. Esse padrão é observado no Brasil e em outros países, reforçando o papel de fatores ocupacionais e comportamentais na distribuição da doença.

Em relação à raça/cor, 76% dos casos ocorreram em indivíduos brancos, possivelmente refletindo a composição demográfica do Rio Grande do Sul, cuja população é majoritariamente branca (78,4%), em contraste com a média nacional (43,5%) ^41^. Ainda assim, a literatura indica maior risco de infecção e gravidade entre populações socialmente vulneráveis, especialmente negras e residentes em periferias ^5^ ^,^ ^6^ ^,^ ^11^, o que exige interpretação cautelosa dos dados e a integração de determinantes socioeconômicos, ambientais e demográficos nas análises epidemiológicas.

Quanto à escolaridade, “Ensino Médio completo” foi a categoria mais frequente (13,3%), embora a elevada proporção de registros ignorados (58,4%) limite análises mais detalhadas. Evidências indicam que baixa escolaridade se associa a piores condições habitacionais, menor acesso à informação e maior exposição ambiental, ampliando o risco de adoecimento ^5^ ^,^ ^42^.

A taxa de letalidade foi de 3,48%, inferior às médias nacional (~9%) e global (~5%) ^11^ ^,^ ^12^, realidade possivelmente relacionada à adoção de critérios de confirmação mais sensíveis ‒ com encerramento por critérios clínico-epidemiológicos e inclusão de formas clínicas mais leves. Contudo, entre indivíduos ≥ 60 anos, a letalidade atingiu 8,26% (10 óbitos em 121 casos), corroborando a literatura que aponta idade avançada e comorbidades como fatores de risco para óbito ^43^.

Os achados indicam que a leptospirose não pode ser compreendida apenas pelo risco ambiental imediato das enchentes. Como doença tropical negligenciada, trata-se de um problema de saúde pública profundamente enraizado em desigualdades socioeconômicas e estruturais, frequentemente permanecendo “invisível” para gestores e tomadores de decisão ^44^. A literatura demonstra maior risco de transmissão em domicílios de baixa renda, associados à presença de roedores, esgoto a céu aberto, acúmulo de lixo e contato frequente com lama contaminada ^6^.

Em contextos de enchentes, essas vulnerabilidades preexistentes são intensificadas, afetando de forma desigual diferentes grupos sociais. Comunidades de menor renda, menor escolaridade e residentes de áreas periféricas e ribeirinhas apresentam maior exposição ^45^, o que amplia o risco de transmissão durante o contato com água de inundação ou lama ^46^. Assim, territórios marcados por pobreza, informalidade urbana e insegurança habitacional concentram a maior carga de impactos, tanto em períodos usuais quanto durante desastres climáticos ^44^.

A sobreposição entre vulnerabilidade social e risco ambiental evidencia que o enfrentamento da leptospirose deve ir além das ações clínicas, incorporando uma abordagem intersetorial de gestão de riscos. Em cenários de enchentes, determinantes estruturais, como saneamento insuficiente, habitação precária e falhas nos sistemas de drenagem, ampliam a exposição a ambientes contaminados e elevam o risco de transmissão ^5^ ^,^ ^6^. No Rio Grande do Sul, apenas 26,6% do esgoto possui tratamento adequado, e as falhas dos sistemas de proteção contra inundações, como rupturas de comportas, refluxos em galerias de drenagem e ineficiência dos diques, durante as enchentes de 2024 evidenciaram a fragilidade da infraestrutura urbana diante de eventos hidrometeorológicos extremos ^9^ ^,^ ^47^.

Esses elementos indicam que o aumento de casos em 2024 não decorre apenas das chuvas intensas, mas da interação entre desigualdades socioespaciais preexistentes e baixa capacidade adaptativa do território. Tal cenário reforça a leptospirose como doença negligenciada e socialmente determinada, cuja prevenção exige tanto respostas emergenciais do SUS quanto investimentos estruturais e de longo prazo em saneamento, urbanização e mitigação de desastres.

A análise espacial da leptospirose no Rio Grande do Sul, representada nos mapas de 2023 e 2024, reforça o geoprocessamento como ferramenta epidemiológica ^48^. Evidências internacionais indicam que enchentes alteram a distribuição geográfica da doença, promovendo expansão territorial e intensificação em áreas previamente afetadas ^13^. A comparação temporal dos mapas permite visualizar a magnitude do surto de 2024 e identificar padrões espaciais sugestivos da associação entre eventos hidrometeorológicos extremos e a dinâmica da leptospirose. Nesse contexto, o uso de sistemas de informação geográfica é amplamente recomendado para o monitoramento de doenças sensíveis ao clima e para a resposta rápida em emergências ^13^ ^,^ ^48^.

Em 2023, os casos concentraram-se nas bacias dos rios Sinos e Caí e do Lago Guaíba. Em 2024, além da intensificação nessas áreas, observou-se expansão para o Vale do Taquari, o Baixo Jacuí e as regiões costeiras de Pelotas e Rio Grande, severamente atingidas pelas enchentes. Esse padrão corrobora a literatura que reconhece os cursos d’água como eixos de difusão da leptospirose em cenários de inundação ^49^. A análise por bacias hidrográficas amplia a interpretação territorial da doença e constitui estratégia-chave para orientar ações preventivas, alocar recursos e fortalecer a vigilância ambiental ^48^.

O aumento de casos durante as enchentes de 2024 reforça a necessidade de estratégias contínuas e integradas de saúde pública. Para além das respostas emergenciais, são fundamentais sistemas de alerta precoce, comunicação de risco eficaz e planos de contingência que viabilizem a rápida mobilização da vigilância e da assistência. Medidas estruturais, como controle de roedores, ampliação do saneamento e aprimoramento do planejamento urbano, devem ser priorizadas em áreas de maior risco ^50^, assim como ações educativas voltadas ao uso seguro da água e à proteção individual em contextos de alagamento ^30^.

No âmbito assistencial, destaca-se a coleta oportuna de amostras para diagnóstico, conforme protocolos estaduais ^39^, associada à antibioticoterapia precoce em pacientes sintomáticos expostos ‒ estratégia central para reduzir a letalidade, especialmente em cenários de sobrecarga hospitalar.

A integração entre saúde pública e planejamento ambiental configura um eixo estratégico de longo prazo ^50^. Evidências internacionais demonstram que o monitoramento climático pode antecipar surtos ‒ como modelos associados ao El Niño que previram até 89% dos eventos na Argentina ^51^ ‒, reforçando que o alinhamento entre vigilância epidemiológica, infraestrutura urbana e monitoramento hidrometeorológico é fundamental para reduzir a vulnerabilidade diante de futuros desastres climáticos.

Por fim, devem ser reconhecidas as limitações do estudo. Não foi possível realizar análises históricas mais robustas, uma vez que apenas 2023 pôde ser utilizado como referência comparativa; os anos anteriores (2020-2022), marcados pela pandemia de COVID-19, apresentaram importante subnotificação de doenças infecciosas, comprometendo a confiabilidade das séries ^52^. Adicionalmente, limitações de qualidade e completude dos bancos públicos restringiram algumas análises: embora a escolaridade estivesse disponível para notificações e óbitos, esse detalhamento não existia para internações, limitando a caracterização sociodemográfica desse desfecho. Por fim, devem ser considerados a subnotificação e os atrasos de registro inerentes aos sistemas de informação em saúde.

## Conclusão

A leptospirose permanece uma doença negligenciada, com baixa visibilidade frente a outros agravos associados a determinantes sociais e ambientais ^4^ ^,^ ^50^, apesar de sua letalidade global estimada em 5,72% ^11^. O aumento de casos observado no Rio Grande do Sul em 2024 evidencia como eventos hidrometeorológicos extremos podem intensificar rapidamente a transmissão, sobrecarregar serviços de saúde e resultar em desfechos graves, incluindo hospitalizações e óbitos ^29^ ^,^ ^30^. Essa experiência reforça a necessidade de incorporar a leptospirose de forma mais explícita nas agendas nacionais e internacionais de saúde pública. Estudos futuros devem incluir abordagens qualitativas que explorem percepções de populações vulneráveis sobre o risco de adoecimento e morte em contextos de desastre, subsidiando estratégias mais eficazes de comunicação em saúde, políticas públicas e promoção da equidade social e ambiental.

## Data Availability

As fontes de informação utilizadas no estudo estão indicadas no corpo do artigo.
